# The Vibriolysin-Like Protease VnpA and the Collagenase ColA Are Required for Full Virulence of the Bivalve Mollusks Pathogen *Vibrio neptunius*

**DOI:** 10.3390/antibiotics10040391

**Published:** 2021-04-06

**Authors:** Fabián Galvis, Juan L. Barja, Manuel L. Lemos, Miguel Balado

**Affiliations:** Departamento de Microbiología y Parasitología, Instituto de Acuicultura y CIBUS-Facultad de Biología, Universidade de Santiago de Compostela, 15705 Santiago de Compostela, A Coruña, Spain; fgs999@hotmail.com (F.G.); juanluis.barja@usc.es (J.L.B.); manuel.lemos@usc.es (M.L.L.)

**Keywords:** *Vibrio neptunius*, virulence, bivalve mollusk pathogen, metalloprotease, collagenase, VnpA, ColA

## Abstract

*Vibrio neptunius* is an important pathogen of bivalve mollusks worldwide. Several metalloproteases have been described as virulence factors in species of *Vibrio* that are pathogenic to bivalves, but little is known about the contribution of these potential virulence factors to *Vibrio neptunius* pathogenesis. In silico analysis of the genome of *V. neptunius* strain PP-145.98 led to the identification of two hitherto uncharacterized chromosomal loci encoding a probable vibriolysin-like metalloprotease and a putative collagenase, which were designated VnpA and ColA, respectively. Single defective mutants of each gene were obtained in *V. neptunius* PP-145.98, and the phospholipase, esterase and collagenase activities were studied and compared with those of the wild-type strain. The results showed that the single inactivation of *vnpA* resulted in a 3-fold reduction in phospholipase/esterase activity. Inactivation of *colA* reduced the collagenase activity by 50%. Finally, infection challenges performed in oyster larvae showed that Δ*vnpA* and Δ*colA*—single mutant strains of *V. neptunius*—are between 2–3-fold less virulent than the wild-type strain. Thus, the present work demonstrates that the production of both VnpA and ColA is required for the full virulence of the bivalve pathogen *V. neptunius*.

## 1. Introduction

Bivalve mollusks are an important source of animal protein for human nutrition, representing about 15% of the average animal protein consumed per capita [1]. The decrease in natural beds has required the establishment of hatcheries to provide farms with juveniles. Unfortunately, the optimum conditions for the growth and development of bivalve larvae in hatcheries favor the occurrence of bacterial infections [2,3].

Several species of the bacterial genus *Vibrio* can cause disease in aquatic animals and/or humans [4,5,6]. Specifically, some species are pathogenic to mollusks and cause vibriosis, which leads to high mortality ratios in larvae and spats of farmed bivalves worldwide [7,8]. *Vibrio* species belonging to the Corallilyticus and Orientalis clades, such as *Vibrio coralliilyticus, V. tubiashii* and *V. neptunius*, are among the best-known species of bivalve pathogens [7]. In particular, *V. neptunius* has been isolated from samples of sea water and marine animals, including turbot larvae (*Scophthalmus maximus*) and rotifers (*Brachiomus plicatilis*) [9,10]. This bacterium has also been reported to be responsible for larval and spat mortality episodes in clam and oyster cultures, leading to significant economic losses in shellfish hatcheries [3]. *V. neptunius* is able to rapidly invade the tissues of bivalve larvae after entering by filtration, and it produces thermolabile extracellular products demonstrating enzymatic activities, such as amylase, gelatinase and lipase, that are cytotoxic to fish and homeothermic animal cell lines [11]. However, the genetic basis of the pathogenicity of this bacterium remains unclear, as there are no known studies on the characterization of genes that encode proteases or other hydrolytic enzymes that might play an active role in the pathogenic character of *V. neptunius*.

Virulence is a multifactorial trait that results from the sequential action of several microbial factors that enable colonization, proliferation in the host and, finally, the infection process [12]. Some metalloproteases, including vibriolysin-like proteases and collagenases with a role in virulence, have been described in vibrios isolated from seawater, fish and shellfish [13,14]. The metalloproteases produced by human pathogenic vibrios have been extensively studied, and the evidence shows a broad range of pathological effects [15,16]. In *V. cholerae*, vibriolysin is a direct toxic factor that causes host tissue damage, forms edematous lesions and facilitates heme utilization [17]. Furthermore, it has indirect toxic effects by converting the precursor of an enterotoxic hemolysin into the mature toxin through propeptide release [18,19], increasing bacterial attachment to the intestinal surface through digestion of the mucosal substances [20] and accelerating bacterial detachment via the digestion of *V. cholerae* adhesins [21]. In *V. coralliilyticus*, one of the most studied pathogens of corals and mollusks, vibriolysin-like metalloproteases encoded by *vcpA* and its orthologs *vcpB* and *vchA* facilitate bacterial invasion and the infection process [22,23]. Recently, an immunoassay to detect VcpA was proposed as a diagnostic tool capable of tracking pathogens involved in coral disease outbreaks [24]. Metalloproteases with roles in virulence have also been described in several bivalve pathogens, such as *V. coralliilyticus* [22,23], *V. tubiashii* [25], *V. aestuarianus* [26] and *V. splendidus* [27]. However, the influence of these proteases on the virulence of different pathogenic bacteria is diverse. On the other hand, collagen is a major component of the extracellular matrix of animal tissues, and thus, collagenase production can also accelerate bacterial dissemination and facilitate nutrient acquisition [28]. Unfortunately, the role of collagenases in the virulence of bivalve pathogens is poorly studied, as most works have focused on the pathogenic effect of collagenase in humans, considering bivalves as reservoirs of bacteria that are pathogenic to humans [29,30].

In this work, an in silico analysis of the genome of *V. neptunius* strain PP-145.98 led to the identification of two hitherto uncharacterized chromosomal loci encoding a probable vibriolysin-like metalloprotease and a putative collagenase, which were designated VnpA and ColA, respectively. The construction of mutants defective in each of these genes resulted in a reduction in the respective phospholipase/esterase and collagenase activities. In addition, infection challenges in oyster larvae showed that the production of both proteases is required for the full virulence of *V. neptunius* in bivalve mollusks. Our results show for the first time that collagenases may be key virulence factors in *Vibrio* species that are pathogenic to bivalve mollusks.

## 2. Materials and Methods

### 2.1. Bacterial Strains, Plasmids and Growth Conditions

The strains and plasmids used, as well as those derived from this study, are included in Table 1. *V. neptunius* strains were grown at 25 °C in Tryptic Soy Agar and Broth (Pronadisa, Madrid, Spain) supplemented with 1% NaCl (TSA-1 and TSB-1, respectively), as well as in M9 minimal medium supplemented with 0.2% Casamino Acids (Difco, Leeuwarden, The Netherlands) (CM9) [31]. The *V. neptunius* strains used to evaluate enzymatic activities belong to the laboratory strain collection. They were isolated from vibriosis outbreaks affecting bivalve hatcheries in Galicia (Spain). *Escherichia coli* strains were grown at 37 °C in Luria–Bertani (LB) medium (Pronadisa) or LB supplemented with the appropriate antibiotics. Ampicillin sodium salt and kanamycin were used at 100 µg mL^−1^ and 50 µg mL^−1^, respectively (final concentrations).

### 2.2. General DNA Manipulation Techniques and PCR Assays

Total genomic DNA from *V. neptunius* was purified with the InstaGene™ Matrix (BioRad, Hercules, California, CA, USA) and stored at −20 °C. The extraction of DNA from agarose gels and purification of plasmid DNA were carried out using NucleoSpin^®^ Gel and a PCR clean-up kit (Macherey-Nagel, Düren, Germany) and a GeneJET Plasmid Miniprep Kit (Thermo-Fisher, Waltham, MA, USA). PCR reactions were all carried out in a T-Gradient Thermal Cycler (Biometra, Göttingen, Germany) with Taq polymerase NZYTaq (Nzytech, Lisboa, Portugal). Thermal cycling conditions were as follow: 95 °C for 5 min, 30 cycles at 95 °C for 45 s, 58 °C for 45 s, and an elongation step at 72 °C for 1 min per kb.

### 2.3. The Search for Candidate Genes and Construction of vnpA and colA Mutants by Allelic Exchange

Candidate genes to encode metalloproteases related to virulence in the genome of *V. neptunius* PP-145.98 (Accession No. JAFHLB000000000) were identified using NCBI services (http://ncbi.nlm.nih.gov, accessed on 1 January 2021). For this purpose, the protein sequences of metalloproteases HapA (Accession No. WP_000782181.1), VcpA (Accession No. AFK08684.1) and ColP (Accession No. WP_069531114.1) were used as queries in the BLAST program to identify homologs in *V. neptunius* PP-145.98. The prediction of protein domains was carried out using the UniProt database facilities (https://www.uniprot.org, accessed on 1 February 2021).

In-frame deletions of *vnpA* and *colA* in *V. neptunius* PP-145.98 were constructed using PCR amplification of two fragments of each gene and the flanking regions, which, when ligated together, should result in an in-frame (nonpolar) deletion. The oligonucleotides used to amplify the upstream and downstream ends of each gene are shown in Table 2. Suitable restriction sites were added to the oligonucleotides. The PCR-amplified 3′-end gene fragments were ligated into pWKS30, and the resulting plasmids were cut with suitable enzymes and ligated to the 5′-ends of the PCR fragments of each gene. Each deleted allele cloned in pWKS30 was digested with NotI and ApaI and ligated into the suicide vector pCAR109 [34]. As a pCVD442 derivative, pCAR109 contains R6K *ori*, requiring the *pir* gene product for replication, and the *sacB* gene, conferring sucrose sensitivity. The resulting plasmids were mated from *E. coli* S17-1-ʎpir into *V. neptunius* PP-145.98, and transformants, in which the plasmid was integrated into the chromosome by homologous recombination, were selected on agar medium containing 50 mg mL^−1^ kanamycin (resistance conferred by pCAR109) and 100 mg mL^−1^ ampicillin (specific antibiotic to select for *V. neptunius* PP-145.98). A second recombination event was obtained by selecting for sucrose (10%) resistance. This process led to the generation of Δ*colA* and Δ*vnpA*, which were named FG56 and FGE5, respectively, as defective mutant strains of *V. neptunius* PP-145.98. Sanger sequencing using primers 1 and 4 (Table 2) was used to confirm mutant construction.

### 2.4. Enzymatic Activity

Enzymatic activities were evaluated out by placing 5 µL of a TSB-1 culture of each strain at OD_600_ ≈ 0.5 on the surface of TSA-1 plates supplemented with the appropriate substrate. All assays were repeated three times to ensure reproducibility of results. TSA-1 plates supplemented with 1% egg yolk were used to determine phospholipase activity [35]. TSA-1 plates with 1% Tween 20 were used to confirm lipolytic activity, as Tween 20 is easily hydrolyzed by esterase because it contains esters of lower chain fatty acids [36]. Both lipolytic activities (phospholipase and esterase) were evaluated after 24 h incubation at 25 °C. The gelatinase activity test was carried out on TSA-1 plates supplemented with 0.4% gelatin, and the results were recorded after 48 h incubation at 25 °C by covering the surface of the agar plate with a 12.5% (*w/v*) HgCl_2_ solution. For the hemolysis test, 5 µL of an inoculum in TSB-1 at OD_600_ ≈ 0.5 was placed on sheep blood agar plates (Oxoid, Hampshire, UK) and incubated at 25 °C for 48 h.

### 2.5. Virulence Assays

Pathogenicity assays were carried out in 6-well sterile plastic cell-culture microplates. Oyster larvae of *Ostrea edulis* were placed in each well with 9 mL of autoclaved seawater at an average density of 11 larvae mL^–1^. Microplates were incubated on an orbital shaker (100 rpm) at room temperature (22 °C). No food was added during the experimental period. The wild-type strain and the mutants Δ*colA* and Δ*vnpA* of *V. neptunius* were cultured in CM9 medium for 12 h at 25 °C to achieve an OD_600_ = 0.5. Bacteria were added to the wells to achieve final concentrations ranging from 10^4^ to 10^5^ cfu mL^–1^. Wells without added bacteria served as negative controls for the assay. Bioassays were performed in triplicate. The larvae were observed under an optical microscope (4× and 10×) to report the effect of the bacteria: live larvae were identified as those that were swimming or had closed valves but showed internal movement, and larvae without internal movement were considered dead. Pathogenicity results were evaluated at 96 h and expressed as a percentage of survival. Mortalities were recorded daily, and the statistical significance of differences in percentage survival for *V. neptunius* strains was determined using the Kaplan–Meier method with the Mantel–Cox log-rank test using SPSS (version 20; IBM SPSS Inc., Chicago, IL, USA). *P*-values were considered significant when *p* was <0.05, <0.01 and <0.001.

## 3. Results

### 3.1. V. neptunius Strains Show Proteolytic and Lipolytic Activities

To assess the extracellular enzymatic activity of *V. neptunius*, a collection of 19 strains isolated from disease episodes that occurred in diverse bivalve larvae was screened in three replicates for proteolytic, lipolytic and hemolytic activities. Most *V. neptunius* isolates were positive for gelatinase, esterase and phospholipase activities (Table 3). The unique exception was strain PP-256, which was considered negative for esterase activity since it failed to hydrolyze Tween 20. In addition, most strains (15 out of 19) showed β-hemolysis, producing a translucent hemolytic halo between 2 and 5 mm wide on sheep blood agar plates. However, two strains (PP-266 and PP-273) were non-hemolytic, and the PP-256 and PP-258 strains showed α-hemolysis since they did not produce translucent halos on sheep blood agar. The results clearly show that all *V. neptunius* strains possess both proteolytic and lipolytic activities. Since PP-145.98 is a *V. neptunius* strain that shows one of the highest proteolytic/lipolytic activities, and its genome was readily available, we chose this strain for an in silico analysis to identify genes that are suspected to encode proteases with a putative role in virulence.

### 3.2. The V. neptunius Genome Contains Genes Encoding Two Metalloproteases Putatively Related to Virulence

**Figure 1:**
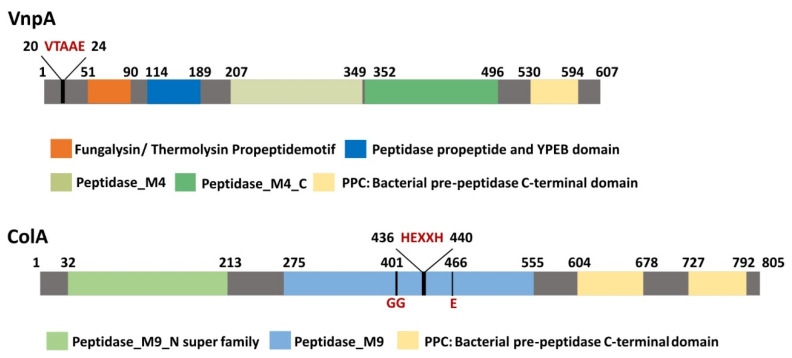
Conserved domains and motifs found in VnpA and ColA metalloproteases.

Many of the toxic proteases produced by vibrios fall into the vibriolysin and collagenase groups, which belong to the metalloprotease family [28]. The in silico analysis of the genome of *V. neptunius* PP-145.98 (Accession No. JAFHLB000000000) showed that it harbors a number of loci encoding putative metalloproteases. According to homology searches, two of them, loci JYA62_21125 of 1821 bp and JYA62_12825 of 2415 bp, are likely to encode virulence-related genes (Table 4). The predicted product of locus JYA62_21125 (protein ID MBN3580162.1) shares close homology (between 74% and 90% similarity) with several vibriolysin-like proteases belonging to the thermolysin family (M4) of metallopeptidases described in other marine *Vibrio* species, including VcpA of *V. coralliilyticus* and VtpA of *V. tubiashii*, proteins that were shown to be major virulence factors of their respective bacterial species [22,37]. Thus, we designated locus JYA62_21125 as *vnpA* (*Vibrio neptunius* metalloprotease A). Notably, VnpA is a homolog of HapA (82% similarity), which is an extracellular Zn-dependent metalloprotease, hemagglutinin/protease produced by *V. cholerae* and also known as vibriolysin [19]. Thus, VnpA is predicted to be a protein of 607 amino acids, containing the characteristic domain architecture conserved between vibriolysins [15,38]. It contains a signal peptide signature in the N-terminal region (between positions 20 and 24 (VTA-AE)), two conserved propeptide motifs between 51 and 189 (FTP domain between 51 and 90 and PepSy domain 114 and 189), mature termolysin matallopeptidase domains (207 and 496) and a C-terminal pre-peptidase (PPC) domain (530 and 594). Conserved domains and motifs found in VnpA metalloproteases are shown in Figure 1.

On the other hand, the predicted product of locus JYA62_12825 (protein ID MBN3578544.1) is a protein of 805 amino acids of which the closest homolog (93% similarity) is a putative collagenase of *V. coralliilyticus* belonging to class II metalloproteinases (Table 4). This protein is predicted to degrade collagen since it contains the conserved zinc-binding motif comprising the sequence HEXXH-E at positions 436 and 466 and a double glycine motif upstream of the HEXXH motif (Figure 1): both domains are required for collagenase activity [39]. These motifs are also present in collagenases from other pathogenic bacteria, such as *Bacillus anthracis*, *B. cereus*, *Clostridium tetani* and *C. botulinum* [40]. Notably, ColA showed significant homology with ColP (39% identity and 60% similarity), a collagenase that was characterized as a major virulence factor of the fish pathogen *Photobacterium damselae* subsp. *damselae* [41]. Thus, locus JYA62_12825 was termed *colA* (collagenase A).

### 3.3. Inactivation of VnpA and ColA Reduced Proteolytic Activity

**Figure 2:**
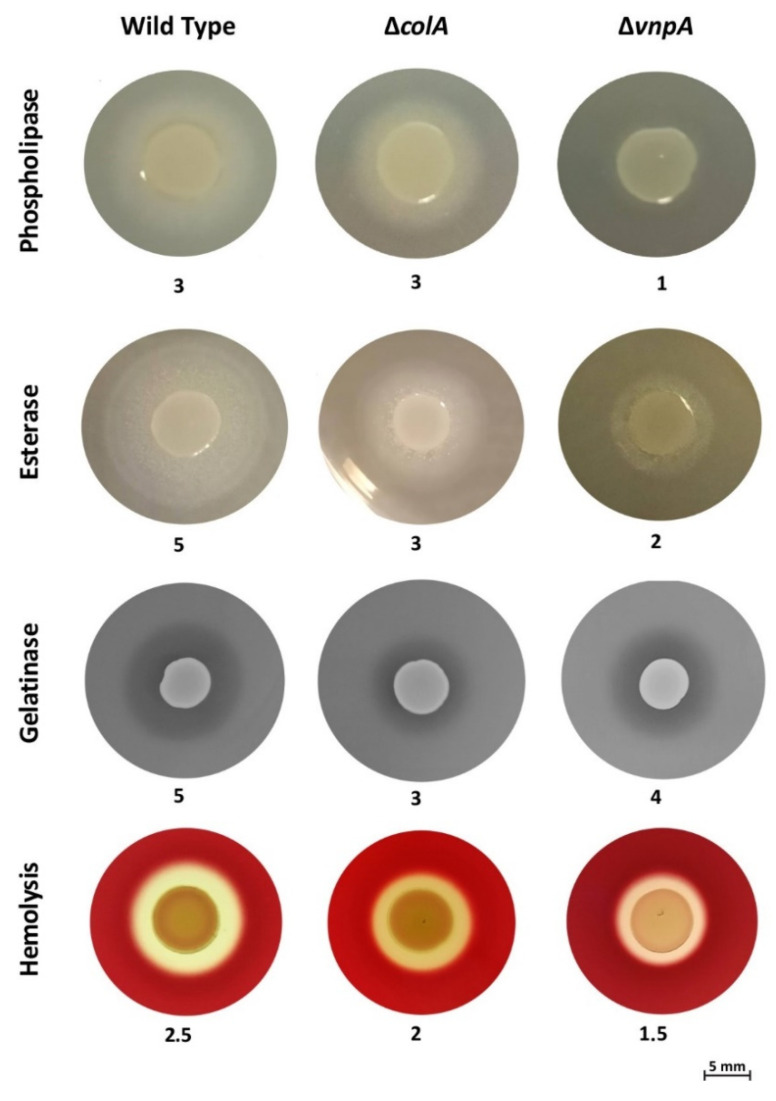
Representative results of phospholipase, esterase, gelatinase and hemolytic activities in the PP-145.98 wild-type strain and in its derivatives *vnpA*- and *colA*-defective mutants. Numbers denote halo sizes in millimeters.

To study the role of *vnpA* and *colA* in the extracellular enzymatic activities and virulence of *V. neptunius*, in-frame deletion mutants of each gene were produced in the *V. neptunius* PP-145.95 background. Then, the ability to hydrolyze egg yolk (phospholipase activity), Tween 20 (esterase activity) and gelatin (gelatinase activity) was evaluated in both mutant strains and compared with those of the wild type (Figure 2). The wild-type *V. neptunius* PP-145.98 strain yielded an opaque precipitation halo in the egg yolk and Tween 20 plates, which is indicative of phospholipase and esterase activities. Interestingly, the Δ*vnpA* mutant was virtually unable to hydrolyze egg yolk and showed a 3-fold reduction in esterase activity. The gelatinase activity of this mutant was almost identical to that of the wild type. Thus, inactivation of *vnpA* in *V. neptunius* disables the phospholipase activity and greatly reduces esterase activity.

When the phenotype of the *V. neptunius* Δ*colA* mutant was evaluated, a major effect on gelatin hydrolysis was detected, as the halo was 2-fold narrower (Figure 2). By contrast, a slight reduction in Tween 20 hydrolysis was observed, and the halo formed in the egg yolk was indistinguishable from that produced by the wild-type strain. In addition, since hemolysis may result from phospholipase enzyme activity [42], the hemolytic phenotype of Δ*vnpA* and Δ*colA* mutants was also evaluated. Interestingly, although the hemolysis produced by the Δ*colA* mutant was the same as that shown by the wild-type strain, the hemolytic halo of the Δ*vnpA* mutant was halved. This result shows that VnpA activity is involved in the lysis of erythrocytes. However, since the Δ*vnpA* mutant retains some degree of hemolytic activity, it is not likely a major hemolytic factor of *V. neptunius*. Thus, inactivation of *vnpA* in *V. neptunius* greatly reduces phospholipase and esterase activities and slightly reduces hemolytic activity. Taken together, the results suggest that VnpA is responsible for the phospholipase and esterase activities in *V. neptunius* and that ColA is a protease that hydrolyzes gelatin.

Screening for the presence of *vnpA* and *colA* by PCR showed that these genes are widespread in *V. neptunius* strains. However, some strains failed in the amplification of the PCR target for either *vnpA* (two strains of 19) or *colA* (one of 19) but had an enzymatic activity profile that was almost identical to that of the PP-145.98 strain (Table 3). Thus, additional proteases must be present in the genome of this strain.

### 3.4. VnpA and ColA Have a Major Role in V. neptunius Virulence

**Figure 3:**
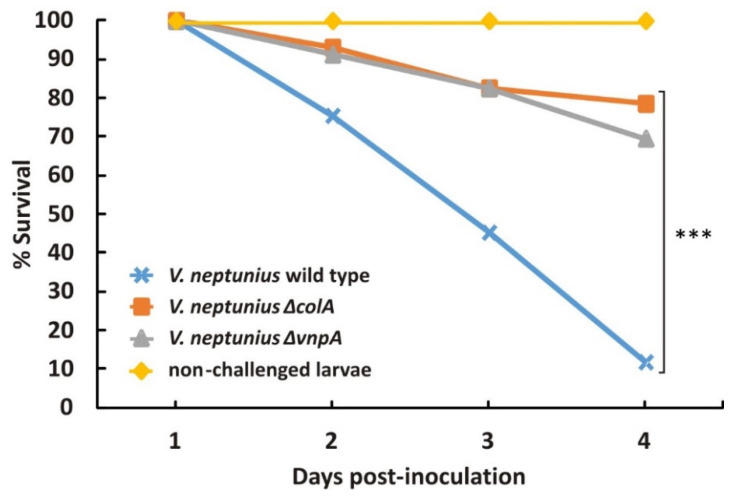
Survival curves after challenge in oyster larvae (*Ostrea edulis*) with the wild-type *V. neptunius* strain or Δ*colA*- or Δ*vnpA*-defective mutants. Asterisks denote statistically significant differences between strains (*** *p* < 0.001).

Proteases can play major roles in colonization and persistence within a host or the degradation of host biomass for nutrient acquisition [28,43]. To study the contribution of VnpA and ColA to *V. neptunius* pathogenesis, the virulence of the PP-145.98 wild-type strain, as well as that of both single Δ*vnpA* and Δ*colA* mutants, was evaluated by performing experimental infections in oyster larvae of *O. edulis*. Infection challenges were performed in triplicate; similar results were obtained in each replicate, and the data were pooled together. The survival curves observed after infection challenges are shown in Figure 3. The mortality was zero in the control group (non-challenged clam larvae). By contrast, inoculation of the PP-145.98 wild-type strain caused high mortality ratios, killing 88% of the group after 96 h. This result was comparable to the mortalities previously reported by Prado et al. for the same strain [3]. Interestingly, the mortality observed after a challenge with either Δ*colA* or Δ*vnpA* mutant strains decreased to 21% and 30%, respectively. In summary, the inactivation of either *colA* or *vnpA* produces a significant reduction in *V. neptunius* virulence, and thus, both genes (*colA* and *vnpA*) are required for the full virulence of *V. neptunius* in oyster larvae.

## 4. Discussion

Vibrionaceae accounts for a dominant proportion of bivalve mollusk microbiota, providing them with a wide range of beneficial services, including defense against external pathogens and facilitation of nutrient acquisition [44,45,46,47,48,49]. However, bacterial inhabitants themselves can act as opportunistic pathogens [50,51]. Notably, the production of certain potential virulence factors, such as siderophores and proteases, is a widespread property of healthy bivalve microbiota [52]. Although siderophores are considered indirect virulence factors that play roles in the establishment of the infection, proteases are usually toxic factors that cause disease symptoms [15].

*V. neptunius* is an opportunistic pathogen in bivalve mollusk microbiota and is responsible for larval and spat mortality episodes in clam and oyster cultures [3,53,54]. In this work, enzymatic activities such as phospholipase, esterase and gelatinase, as well as hemolysis, were evaluated using a collection of 19 *V. neptunius* strains isolated from outbreak episodes affecting oysters and clams. The results show that *V. neptunius* displays lipolytic and proteolytic activities, with some degree of variability among strains. The same strain collection was used in a recent work to study siderophore production, and major differences among isolates were also detected [55]. The absence of diversity usually indicates a rapid spread of pandemic strains [56]. In contrast, the results obtained in this work suggest a long-term association between *V. neptunius* and bivalves, which supports the idea that *V. neptunius* is an endemic opportunistic pathogen present in mollusk ecosystems [3]. The optimum conditions for the growth and development of bivalve larvae in hatcheries (densities, temperature, load organic matter, etc.) favor the multiplication of bacteria and the occurrence of bacterial infections [2,3].

Extracellular metalloproteases, including vibriolysins and collagenases, are widely distributed among commensal and pathogenic species of *Vibrio* [13,14,28]. In this work, two hitherto uncharacterized chromosomal loci encoding a vibriolysin-like protease and a probable collagenase were identified in the genome of *V. neptunius* strain PP-145.98 and were designated *vnpA* and *colA*, respectively. The phenotype of the *V. neptunius* Δ*vnpA* mutant strongly suggests that VnpA is the main enzyme responsible for the lipolytic activity of this bacterium. VnpA shares high similarity with vibriolysin-like proteases with phospholipase activity from a variety of bivalve pathogens, such as *V. aestuarianus* [26], *V. tubiashii* [25], *V coralliilyticus* [22] and *V. splendidus* [27]. Interestingly, the mutation of *vnpA* also induced a slight reduction in the gelatinase and hemolytic activity of *V. neptunius*. These results are congruent with those found in *V. coralliiilyticus*, where inactivation of *vcpA*, a close homolog of *vnpA*, produced a significant reduction in phospholipase and collagenase activities [22]. *V. neptunius* ColA contains the catalytic domain structure that is conserved among collagenases, and its defective mutant strain showed reduced gelatinase activity. Gelatin is frequently used as a substrate to detect proteases, including collagenases [57]. Thus, collectively, all results strongly suggest that *colA* encodes a collagenase.

Virulence is a context-dependent multifactorial trait, where the contribution of a particular virulence factor depends on the genetic repertoire of a specific pathogen and on host–pathogen interactions [58]. The results obtained from infection challenges strongly suggest that the genes analyzed, namely, *vnpA* and *colA*, are required for the full virulence of *V. neptunius*. While vibriolysin-like proteases can play multiple roles in pathogenesis [15,16], collagenases can be involved in colonization and proliferation in the host tissues [59]. Mutagenesis of *vnpA* homologs in *V. tubiashii* and *V. splendidus* was also associated with a significant reduction in lethality in oysters [27,60,61]. By contrast, in *V. coralliilyticus*, the single inactivation of *vcpA* did not appear to reduce virulence [22]. Notably, the genome of *V. coralliilyticus* harbors three *vcpA* paralogs named *vcpA*, *vcpB* and *vchA* [23]. Thus, it is tempting to hypothesize that differing contributions to virulence are based on the existence of gene duplications (paralogs). However, since *V. tubiashii* also harbors three paralogs of these genes (*vtpA*, *vtpB* and *vthA*) [23], this hypothesis is not fully sustainable. Bacterial pathogens are highly specific in their infection behavior against different host organisms [62]. Thus, a plausible explanation relies on the fact that virulence challenges reported for the *V. coralliilyticus vcpA*-defective mutant were performed using *Drosophila* and *Artemia* as animal models, and the results might not be comparable [23]. Notably, our results also showed that inactivation of *vnpA* led to a decrease in hemolytic activity, an effect that was also reported in *V. tubiashii* after inactivation of *vtpA* [61]. By contrast, *vcpA* inactivation in *V. coralliilyticus* resulted in a significant increase in hemolytic activity [22]. In this regard, the contribution to virulence of hemolysins from the fish pathogen *Photobacterium damselae* subsp. *damselae* is dependent on their combined expression with damselysin, a metalloprotease with phospholipase activity that has a synergistic effect on virulence, depending on the animal infected [63].

A significant number of collagenolytic organisms associated with pathogenic processes that involve the destruction of collagen-containing tissues have been described [64]. Notably, to date, no known studies have evaluated the contribution of collagenases to the virulence of bivalve pathogens. Inactivation of *colA* in *V. neptunius* significantly reduced the mortality of oyster larvae with respect to the wild-type strain. This result strongly suggests that ColA is required for the full virulence of this species. Several collagenases isolated from various species of *Vibrio* (*V. alginolyticus*, *V. anguillarum*, *V. cholerae*, *V. fluvialis*, *V. parahaemolyticus*, *V. proteolyticus* and *V. vulnificus*) have been postulated as probable virulence factors [64]. However, their role in host colonization is still unclear. In *V. alginolyticus*, the *colA* mutant showed no collagenolytic activity, but no virulence assays were performed [29]. Collagenases of *Clostridium perfringens*, *V. parahaemolyticus* and *Fusobacterium nucleatum* act as virulence factors for tissue invasion and damage [65,66,67]. In addition, collagenase inactivation in *Leptospira* sp. results in a decrease in virulence for mice [59]. Recently, deletion of the *colP* gene reduced the virulence of *P. damselae* subsp. *damselae* in sea bass [41].

## 5. Conclusions

This work shows that *vnpA* encodes a gene that is largely responsible for the phospholipase and esterase activities in *V. neptunius* and that *colA* encodes a collagenase. The results show that both *vnpA* and *colA* are required for the full virulence of *V. neptunius* in larvae of *Ostrea edulis*. Although some vibriolysin-like metalloproteases with a role in virulence were previously characterized in some pathogens of bivalves, our work provides the first evidence of the role of a collagenase in the virulence of this type of pathogen. In addition, some degree of variability in enzymatic activity phenotypes and in *vnpA* and *colA* distribution was observed among *V. neptunius* strains. However, it is currently unknown whether this phenotypic variability correlates with virulence. Further work must be performed to study the geographical distribution of *V. neptunius* and its implications for virulence.

## Figures and Tables

**Table 1:** Strains and plasmids used in this study.

Strain or Plasmid	Relevant Characteristic (s)	Reference or Source
*Vibrio neptunius*		
PP-145.98	Wild-type strain, isolated from *Ruditapes philippinarum* (larvae), Ap^r^	[3]
FG56	PP-145.98 *vnpA* defective mutant, Ap^r^	This study
FGE5	PP-145.98 *colA* defective mutant, Ap^r^	This study
*E. coli*		
DH5α	SupE4 ∆lacU169 (Φ80 lacZ∆M15) hsd R17 recA1 endA1 gyrA96 thi-1 relA1	Laboratory stock
S17-1-λpir	Tp^r^ Smr recA, thi, pro, hsdR-M+RP4: 2- Tc:Mu:Km Tn7 λpir	[32]
Plasmids		
pWKS30	Low-copy-number cloning vector, Ap^r^	[33]
pNidKan	Suicide vector derived from pCVD442, pir, Km^r^	[34]

**Table 2:** Oligonucleotides used for the construction of mutants. Recognition sequences for restriction enzymes are underlined.

Oligonucleotide	Sequence (5′→3′)	Size (bp)
***vnpA*-defective mutant construction**		
1_1105 EcoRI_1 *	CGGGAATTCGCCATTTATCGCTGAAGCAG	1008
1_1105 BamHI_2	GGCGGATCCTACTTTAACCATCTCTGCGG	
1_1105 BamHI_3	CGCGGATCCGCAGGCAGCAAACCCACAAC	1006
1_1105 XbaI_4 *	CGCTCTAGACGCGATTGGATTGGTCACCG	
***colA*-defective mutant construction**		
1_2110 XbaI_1 *	CGCTCTAGAGGCAAGAGTGCCAATGGCGT	991
1_2110 BamHI_2	GGCGGATCCATCCGCGACTTTGACTTGTT	
1_2110 BamHI_3	GGCGGATCCGATGTGTTTGTCGGTCAACA	999
1_2110 EcoRI_4 *	CGGGAATTCGCGTTTCATTTCTGCCTGCA	

* Primers used to verify mutant construction by Sanger sequencing.

**Table 3:** Extracellular hydrolytic activities and the presence of *colA* and *vnpA* genes in a collection of *V. neptunius* strains.

		Activity ^b^					
*V. neptunius* Strain	Origin ^a^	Gelatinase	Esterase	Phospholipase	Hemolysis ^c^	*colA* ^d^	*vnpA* ^d^
PP-145.98	Oe	+++	+++	++	+	+	+
PP-255	Rp	+++	++	++	+++	+	−
PP-256	Rp	+++	−	+	(+)	+	+
PP-258	Rp	+	++	++	(+)	+	+
PP-259	Rp	++	++	++	+	+	+
PP-266	Rp	++	+++	++	−	+	+
PP-267	Rp	++	++	++	++	+	−
PP-269	Rp	++	++	++	++	+	+
PP-273	Rp	++	++	++	−	+	+
PP-302	Oe	++	++	++	++	+	−
PP-307	Oe	++	+++	++	++	+	+
PP-309	Oe	++	++	++	++	+	−
PP-312	Oe	++	++	++	++	−	+
PP-313	Oe	++	++	++	++	+	+
PP-315	Oe	++	++	++	+	+	+
PP-322	Oe	++	++	++	+	+	+
PP-323	Oe	++	++	++	+	+	+
PP-325	Oe	++	++	++	++	+	+
PP-326	Oe	++	++	++	+	+	+

^a^ Isolated from *Ostrea edulis* (Oe) or *Ruditapes philippinarum* (Rp). ^b^ Hydrolytic activities are indicated as the size of clear/opalescent halos: +, small halo ≤ 2 mm; ++, medium halo between 2 and 5 mm; +++, large halo ≥ 5 mm; −, absence of halo. ^c^ Hemolysis is denoted by the size of the β-hemolytic halo: +, small halo ≤ 2 mm; ++, medium halo between 2 and 5 mm; +++, large halo ≥ 5 mm; (+), (α-hemolysis); −, no hemolysis detected. ^d^ Presence (+) or absence (−) of *colA* and *vnpA* genes tested by PCR.

**Table 4:** Significant homologs of VnpA and ColA.

Species (Accession Number)	% Identity	% Similarity	Function
VnpA (607 residues)			
* Vibrio coralliilyticus * (WP_043010653.1)	93	97	metallopeptidase VcpA
* Vibrio tubiashii * (WP_171320543.1)	74	86	metallopeptidase VtpA
* Vibrio cholerae * (WP_142622324.1)	69	82	hemagglutinin/proteinase HapA
ColA (805 residues)			
* Vibrio coralliilyticus * (WP_043010842.1)	88	93	putative collagenase
* Vibrio tubiashii * (AIW15753.1)	57	73	peptidase M9
* Photobacterim damselae * subsp *damselae* (WP_069531114.1)	39	60	collagenase ColP

## Data Availability

The data for this manuscript is available from correspondence author.

## References

[1] FAO (2020). The State of World Fishery and Aquaculture 2020 (SOFIA).

[2] López B.D., Methion S. (2017). The impact of shellfish farming on common bottlenose dolphins’ use of habitat. Mar. Biol..

[3] Prado S., Romalde J.L., Montes J., Barja J.L. (2005). Pathogenic bacteria isolated from disease outbreaks in shellfish hatcheries. First description of *Vibrio neptunius* as an oyster pathogen. Dis. Aquat. Org..

[4] Pruzzo C., Huq A., Colwell R.R., Donelli G. (2006). Pathogenic Vibrio Species in the Marine and Estuarine Environment. Oceans and Health: Pathogens in the Marine Environment.

[5] Austin B. (2010). Vibrios as causal agents of zoonoses. Vet. Microbiol..

[6] Gorrasi S., Pasqualetti M., Franzetti A., Pittino F., Fenice M. (2020). Vibrio communities along a salinity gradient within a marine saltern hypersaline environment (Saline di Tarquinia, Italy). Environ. Microbiol..

[7] Dubert J., Barja J.L., Romalde J.L. (2017). New Insights into Pathogenic Vibrios Affecting Bivalves in Hatcheries: Present and Future Prospects. Front. Microbiol..

[8] Travers M.-A., Miller K.B., Roque A., Friedman C.S. (2015). Bacterial diseases in marine bivalves. J. Invertebr. Pathol..

[9] García-Amado M.A., Bozo-Hurtado L., Astor Y., Suárez P., Chistoserdov A. (2011). Denaturing gradient gel electrophoresis analyses of the vertical distribution and diversity of Vibrio spp. populations in the Cariaco Basin. FEMS Microbiol. Ecol..

[10] Thompson F.L., Li Y., Gomez-Gil B., Thompson C.C., Hoste B., Vandemeulebroecke K., Rupp G.S., Pereira A., De Bem M.M., Sorgeloos P. (2003). *Vibrio neptunius* sp. nov., *Vibrio brasiliensis* sp. nov. and Vibrio xuii sp. nov., isolated from the marine aquaculture environment (bivalves, fish, rotifers and shrimps). Int. J. Syst. Evol. Microbiol..

[11] Dubert J., Nelson D.R., Spinard E.J., Kessner L., Gomez-Chiarri M., Da Costa F., Prado S., Barja J.L. (2016). Following the infection process of vibriosis in Manila clam (*Ruditapes philippinarum*) larvae through GFP-tagged pathogenic Vibrio species. J. Invertebr. Pathol..

[12] Casadevall A., Pirofski L.-A. (2009). Virulence factors and their mechanisms of action: The view from a damage–response framework. J. Water Health.

[13] Huang J., Zeng B., Liu D., Wu R., Zhang J., Liao B., He H., Bian F. (2018). Classification and structural insight into vibriolysin-like proteases of Vibrio pathogenicity. Microb. Pathog..

[14] Saulnier D., De Decker S., Haffner P., Cobret L., Robert M., Garcia C. (2010). A Large-Scale Epidemiological Study to Identify Bacteria Pathogenic to Pacific Oyster Crassostrea gigas and Correlation between Virulence and Metalloprotease-like Activity. Microb. Ecol..

[15] Miyoshi S.-I., Shinoda S. (2000). Microbial metalloproteases and pathogenesis. Microbes Infect..

[16] Shinoda S., Miyoshi S.-I. (2011). Proteases Produced by Vibrios. Biocontrol Sci..

[17] Nishina Y., Miyoshi S., Nagase A., Shinoda S. (1992). Significant role of an exocellular protease in utilization of heme by Vibrio vulnificus. Infect. Immun..

[18] Nagamune K., Yamamoto K., Naka A., Matsuyama J., Miwatani T., Honda T. (1996). In vitro proteolytic processing and activation of the recombinant precursor of El Tor cytolysin/hemolysin (pro-HlyA) of *Vibrio cholerae* by soluble hemagglutinin/protease of V. cholerae, trypsin, and other proteases. Infect. Immun..

[19] Silva A.J., Leitch G.J., Camilli A., Benitez J.A. (2006). Contribution of Hemagglutinin/Protease and Motility to the Pathogenesis of El Tor Biotype Cholera. Infect. Immun..

[20] Alam M., Miyoshi S.-I., Shinoda S. (1995). Production of Antigenically Related Exocellular Elastolytic Proteases Mediating Hemagglutination by Vibrios. Microbiol. Immunol..

[21] Finkelstein R.A., Boesman-Finkelstein M., Chang Y., Häse C.C. (1992). Vibrio cholerae hemagglutinin/protease, colonial variation, virulence, and detachment. Infect. Immun..

[22] De Santos E.O., Alves N.T., Dias G.M., Mazotto A.M., Vermelho A.B., Vora G.J., Wilson B., Beltran V.H., Bourne D.G., Le Roux F. (2011). Genomic and proteomic analyses of the coral pathogen *Vibrio coralliilyticus* reveal a diverse virulence repertoire. ISME J..

[23] Zhao W., Yuan T., Piva C., Spinard E.J., Schuttert C.W., Rowley D.C., Nelson D.R. (2018). The Probiotic BacteriumPhaeobacter inhibensDownregulates Virulence Factor Transcription in the Shellfish Pathogen *Vibrio coralliilyticus* by N-Acyl Homoserine Lactone Production. Appl. Environ. Microbiol..

[24] Ushijima B., Meyer J.L., Thompson S., Pitts K., Marusich M.F., Tittl J., Weatherup E., Reu J., Wetzell R., Aeby G.S. (2020). Disease Diagnostics and Potential Coinfections by *Vibrio coralliilyticus* During an Ongoing Coral Disease Outbreak in Florida. Front. Microbiol..

[25] Delston R.B., Kothary M.H., Shangraw K.A., Tall B.D. (2003). Isolation and characterization of a zinc-containing metalloprotease expressed by *Vibrio tubiashii*. Can. J. Microbiol..

[26] Labreuche Y., Le Roux F., Henry J., Zatylny C., Huvet A., Lambert C., Soudant P., Mazel D., Nicolas J.-L. (2010). Vibrio aestuarianus zinc metalloprotease causes lethality in the Pacific oyster *Ostrea edulis* and impairs the host cellular immune defenses. Fish Shellfish. Immunol..

[27] Le Roux F., Binesse J., Saulnier D., Mazel D. (2006). Construction of a *Vibrio splendidus* Mutant Lacking the Metalloprotease Gene *vsm* by Use of a Novel Counterselectable Suicide Vector. Appl. Environ. Microbiol..

[28] Emiyoshi S.-I. (2013). Extracellular proteolytic enzymes produced by human pathogenic vibrio species. Front. Microbiol..

[29] Mima T., Gotoh K., Yamamoto Y., Maeda K., Shirakawa T., Matsui S., Murata Y., Koide T., Tokumitsu H., Matsushita O. (2017). Expression of Collagenase is Regulated by the VarS/VarA Two-Component Regulatory System in *Vibrio alginolyticus*. J. Membr. Biol..

[30] Dahanayake P.S., Hossain S., Wickramanayake M., Wimalasena S., Heo G. (2019). Manila clam (*Ruditapes philippinarum*) marketed in Korea as a source of vibrios harbouring virulence and β-lactam resistance genes. Lett. Appl. Microbiol..

[31] Lemos M.L., Salinas P., Toranzo A.E., Barja J.L., Crosa J.H. (1988). Chromosome-mediated iron uptake system in pathogenic strains of Vibrio anguillarum. J. Bacteriol..

[32] Herrero M., De Lorenzo V., Timmis K.N. (1990). Transposon vectors containing non-antibiotic resistance selection markers for cloning and stable chromosomal insertion of foreign genes in gram-negative bacteria. J. Bacteriol..

[33] Wang R.F., Kushner S.R. (1991). Construction of versatile low-copy-number vectors for cloning, sequencing and gene expression in Escherichia coli. Gene.

[34] Mouriño S., Osorio C.R., Lemos M.L. (2004). Characterization of Heme Uptake Cluster Genes in the Fish Pathogen *Vibrio anguillarum*. J. Bacteriol..

[35] López-Romalde S., Magariños B., Nunez S., Toranzo A.E., Romalde J.L. (2003). Phenotypic and Genetic Characterization of *Pseudomonas* anguilliseptica Strains Isolated from Fish. J. Aquat. Anim. Health.

[36] Lee L.P., Karbul H.M., Citartan M., Gopinath S.C.B., Lakshmipriya T., Tang T.-H. (2015). Lipase-SecretingBacillusSpecies in an Oil-Contaminated Habitat: Promising Strains to Alleviate Oil Pollution. BioMed Res. Int..

[37] Mersni-Achour R., Ben Cheikh Y., Pichereau V., Doghri I., Etien C., Dégremont L., Saulnier D., Fruitier-Arnaudin I., Travers M.-A. (2015). Factors other than metalloprotease are required for full virulence of French Vibrio tubiashii isolates in oyster larvae. Microbiology.

[38] Miyoshi S., Wakae H., Tomochika K., Shinoda S. (1997). Functional domains of a zinc metalloprotease from Vibrio vulnificus. J. Bacteriol..

[39] Duarte A.S., Correia A., Esteves A.C. (2016). Bacterial collagenases—A review. Crit. Rev. Microbiol..

[40] Abfalter C.M., Schönauer E., Ponnuraj K., Huemer M., Gadermaier G., Regl C., Briza P., Ferreira F., Huber C.G., Brandstetter H. (2016). Cloning, Purification and Characterization of the Collagenase ColA Expressed by *Bacillus cereus* ATCC 14579. PLoS ONE.

[41] Vences A., Rivas A.J., Lemos M.L., Husmann M., Osorio C.R. (2017). Chromosome-Encoded Hemolysin, Phospholipase, and Collagenase in Plasmidless Isolates of *Photobacterium damselae* subsp. *damselae* Contribute to Virulence for Fish. Appl. Environ. Microbiol..

[42] Rivas A.J., Balado M., Lemos M.L., Osorio C.R. (2011). The Photobacterium damselae subsp. damselae Hemolysins Damselysin and HlyA Are Encoded within a New Virulence Plasmid. Infect. Immun..

[43] Johnson C.N. (2013). Fitness Factors in Vibrios: A Mini-review. Microb. Ecol..

[44] McFall-Ngai M., Hadfield M.G., Bosch T.C.G., Carey H.V., Domazet-Lošo T., Douglas A.E., Dubilier N., Eberl G., Fukami T., Gilbert S.F. (2013). Animals in a bacterial world, a new imperative for the life sciences. Proc. Natl. Acad. Sci. USA.

[45] Rosenberg E., Koren O., Reshef L., Efrony R., Zilber-Rosenberg I. (2007). The role of microorganisms in coral health, disease and evolution. Nat. Rev. Genet..

[46] Engel S., Jensen P.R., Fenical W. (2002). Chemical Ecology of Marine Microbial Defense. J. Chem. Ecol..

[47] Desriac F., Le Chevalier P., Brillet B., Leguerinel I., Thuillier B., Paillard C., Fleury Y. (2013). Exploring the hologenome concept in marine bivalvia: Haemolymph microbiota as a pertinent source of probiotics for aquaculture. FEMS Microbiol. Lett..

[48] Defer D., Desriac F., Henry J., Bourgougnon N., Baudy-Floc’H M., Brillet B., Le Chevalier P., Fleury Y. (2013). Antimicrobial peptides in oyster hemolymph: The bacterial connection. Fish Shellfish. Immunol..

[49] Vezzulli L., Stagnaro L., Grande C., Tassistro G., Canesi L., Pruzzo C. (2017). Comparative 16SrDNA Gene-Based Microbiota Profiles of the Pacific Oyster (*Crassostrea gigas*) and the Mediterranean Mussel (*Mytilus galloprovincialis*) from a Shellfish Farm (Ligurian Sea, Italy). Microb. Ecol..

[50] Garnier M., Labreuche Y., Garcia C., Robert M., Nicolas J.-L. (2007). Evidence for the Involvement of Pathogenic Bacteria in Summer Mortalities of the Pacific Oyster Crassostrea gigas. Microb. Ecol..

[51] Cerf-Bensussan N., Gaboriau-Routhiau V. (2010). The immune system and the gut microbiota: Friends or foes?. Nat. Rev. Immunol..

[52] Leite L., Jude-Lemeilleur F., Raymond N., Henriques I., Garabetian F., Alves A. (2017). Phylogenetic diversity and functional characterization of the Manila clam microbiota: A culture-based approach. Environ. Sci. Pollut. Res..

[53] Kesarcodi-Watson A., Kaspar H., Lategan M., Gibson L. (2009). Challenge of New Zealand Greenshell™ mussel *Perna canaliculus* larvae using two Vibrio pathogens: A hatchery study. Dis. Aquat. Org..

[54] Kesarcodi-Watson A., Kaspar H., Lategan M.J., Gibson L. (2009). Two pathogens of Greenshell^TM^ mussel larvae, *Perna Canaliculus*: *Vibrio splendidus* and a *V. coralliilyticus/neptunius*-like isolate. J. Fish Dis..

[55] Galvis F., Ageitos L., Martínez-Matamoros D., Barja J.L., Rodríguez J., Lemos M.L., Jiménez C., Balado M. (2020). The marine bivalve molluscs pathogen *Vibrio neptunius* produces the siderophore amphibactin, which is widespread in molluscs microbiota. Environ. Microbiol..

[56] Pollock F.J., Wilson B., Johnson W.R., Morris P.J., Willis B.L., Bourne D.G. (2010). Phylogeny of the coral pathogen Vibrio coralliilyticus. Environ. Microbiol. Rep..

[57] Salamone M., Nicosia A., Ghersi G., Tagliavia M. (2019). Vibrio Proteases for Biomedical Applications: Modulating the Proteolytic Secretome of *V. alginolyticus* and *V. parahaemolyticus* for Improved Enzymes Production. Microorganisms.

[58] Granato E.T., Harrison F., Kümmerli R., Ross-Gillespie A. (2016). Do Bacterial “Virulence Factors” Always Increase Virulence? A Meta-Analysis of Pyoverdine Production in Pseudomonas aeruginosa As a Test Case. Front. Microbiol..

[59] Kassegne K., Hu W., Ojcius D.M., Sun D., Ge Y., Zhao J., Yang X.F., Li L., Yan J. (2014). Identification of Collagenase as a Critical Virulence Factor for Invasiveness and Transmission of Pathogenic Leptospira Species. J. Infect. Dis..

[60] Binesse J., Delsert C., Saulnier D., Champomier-Vergès M.-C., Zagorec M., Munier-Lehmann H., Mazel D., Le Roux F. (2008). Metalloprotease Vsm Is the Major Determinant of Toxicity for Extracellular Products of Vibrio splendidus. Appl. Environ. Microbiol..

[61] Hasegawa H., Lind E.J., Boin M.A., Häse C.C. (2008). The Extracellular Metalloprotease of Vibrio tubiashii Is a Major Virulence Factor for Pacific Oyster (*Crassostrea gigas*) Larvae. Appl. Environ. Microbiol..

[62] Dandekar T., Eisenreich W. (2015). Host-adapted metabolism and its regulation in bacterial pathogens. Front. Cell. Infect. Microbiol..

[63] Rivas A.J., Balado M., Lemos M.L., Osorio C.R. (2013). Synergistic and Additive Effects of Chromosomal and Plasmid-Encoded Hemolysins Contribute to Hemolysis and Virulence in Photobacterium damselae subsp. damselae. Infect. Immun..

[64] Lee J.-H., Ahn S.-H., Lee E.-M., Jeong S.-H., Kim Y.-O., Lee S.-J., Kong I.-S. (2005). The FAXWXXT motif in the carboxyl terminus ofVibrio mimicusmetalloprotease is involved in binding to collagen. FEBS Lett..

[65] Awad M.M., Ellemor D.M., E Bryant A., Matsushita O., Boyd R.L., Stevens D.L., Emmins J.J., Rood J.I. (2000). Construction and virulence testing of a collagenase mutant of Clostridium perfringens. Microb. Pathog..

[66] Uitto V.-J., Baillie D., Wu Q., Gendron R., Grenier D., Putnins E.E., Kanervo A., Firth J.D. (2005). Fusobacterium nucleatum Increases Collagenase 3 Production and Migration of Epithelial Cells. Infect. Immun..

[67] Miyoshi S.-I., Nitanda Y., Fujii K., Kawahara K., Li T., Maehara Y., Ramamurthy T., Takeda Y., Shinoda S. (2008). Differential gene expression and extracellular secretion of the collagenolytic enzymes by the pathogen Vibrio parahaemolyticus. FEMS Microbiol. Lett..

